# Measurement of intestinal permeability using lactulose and mannitol with conventional five hours and shortened two hours urine collection by two different methods: HPAE-PAD and LC-MSMS

**DOI:** 10.1371/journal.pone.0220397

**Published:** 2019-08-08

**Authors:** Md. Abu Musa, Mamun Kabir, Md. Iqbal Hossain, Emtiaz Ahmed, Abdullah Siddique, Humaira Rashid, Mustafa Mahfuz, Dinesh Mondal, Tahmeed Ahmed, William A. Petri, Rashidul Haque

**Affiliations:** 1 Emerging Infections and Parasitology Laboratory, Infectious Diseases Division, International Centre for Diarrhoeal Disease Research, Bangladesh, Dhaka, Bangladesh; 2 Nutrition and Clinical Services Division, International Centre for Diarrhoeal Disease Research, Bangladesh, Dhaka, Bangladesh; 3 Division of Infectious Diseases and International Health, Department of Medicine, University of Virginia, Charlottesville, Virginia, United States of America; University College London Hospitals NHS Foundation Trust, UNITED KINGDOM

## Abstract

Urinary excretion of two orally-administered non-metabolizable sugars, lactulose and mannitol, is a valuable marker for evaluating intestinal permeability. Usually this test involves a time consuming procedure of about 5 hour’s urine collection, which makes the test incompatible to some extent. As the results are expressed as the ratio of lactulose and mannitol recovered in urine within certain time, it may be possible to get similar result despite the reduced urine collection time of 2 hours. Moreover, different laboratories do the test by different methods, which make the results incomparable between laboratories. Here, we are also trying to find the correlation between results from most commonly used methods: HPAE-PAD and LC-MSMS. The lactulose: mannitol (LM) test was performed in a cohort of Bangladeshi infants considered at-risk for environmental enteropathy. 208 urine specimens from 104 (52 male and 52 female) infants were collected at 2 and 5 hours after LM solution administration and were tested for lactulose and mannitol by two different methods, one HPAE-PAD platform and another LC-MSMS platform. Median age of the children was 15.0 months (range 6.9 to 25.8 months) and their mean weight-for-age z-score was -0.92. A higher percentage of lactulose and mannitol recovery was found in 5 hours urine collection than in the corresponding 2 hours by both HPAE-PAD and LC-MSMS method, but when results were expressed as lactulose to mannitol ratio (LMR) there was no significant difference between 2 and 5 hours urine collection in both HPAE-PAD (P = 0.138) and LC-MSMS (P = 0.099) method. LMR based on 2 hours urine collection correlated well with LMR based on traditional 5 hours urine collection (Spearman’s correlation coefficient 0.578 and 0.604 respectively for HPAE-PAD and LC-MSMS). In future, LM test to assess intestinal permeability in children can be simplified by shortening the urine collection time from 5 hours to 2 hours.

## Introduction

The lactulose: mannitol (LM) test is a quantitative assay for directly measuring the ability of two non-metabolized sugar molecules—lactulose and mannitol—to permeate the intestinal mucosa. Manntiol, a readily-absorbed monomer, serves as a marker of trans-cellular uptake; and lactulose, a dimer, is only slightly-absorbed and serves as a marker for mucosal integrity [1]. An elevated lactulose to mannitol ratio is an indicator of intestinal barrier dysfunction. The LM test can be used as a noninvasive diagnostic aid to detect intestinal barrier dysfunction in patients with diarrheal diseases [2], malnutrition [3], HIV infection [4, 5], trauma [6], surgical stress [7], celiac disease [8], Crohn’s disease [9], active inflammatory bowel disease (IBD), irritable bowel syndrome (IBS) [10], allergy and intolerance to cow’s milk [11, 12], lactase deficiency, mucositis induced by chemotherapy or inflammatory agents [13], as well as a sensitive parameter to follow nutrition or sanitation related intervention studies to assess gastrointestinal health of children living in poverty [13–16].

In spite of the test’s immense potentials, application of the test in clinical research remains limited due to variation in the methodologies such as: study population, sugar solution administration, urine collection time, and assay method between studies. Besides that, urine is collected for 5 to 6 hours after ingestion of sugar solution; this longer collection time makes the test incompatible to some extent. In this study we aimed to compare prolonged 5 hours urine collection and 2 hours urine collection for LM test to estimate the intestinal permeability in children.

## Materials and methods

In the study, 104 children (52 male and 52 female) from the ongoing malnutrition and enteric diseases (MAL-ED) study were randomly included. The study protocol was approved by the institutional review board of icddr,b. The approved protocol number was PR 2008–020. An informed consent paper was signed by each participant or his/her guardians. The children were apparently healthy. Any children suffering from any diarrheal or fever illnesses, congenital defect were excluded from this study. Median age of the children that participated was 15.0 months (range 6.9 to 25.8 months). Fasted children (at least 2 hours) were instructed to drink a solution containing lactulose (250 mg/ml, Osmolax, Square Pharmaceutical Ltd, Dhaka, Bangladesh) and mannitol (50 mg/ml, Sigma, St. Louis, MO, USA) in a dose of 2 ml/Kg of body weight, or a maximum 20 ml. The children were requested to empty their bladders before ingesting the test sugar solution. A pediatric urine collection bag (Fisher cat. #22275347 or Hollister U-bag #7501) was attached to the children to collect their urine. The children were allowed to return to their regular diet 30 minutes after ingestion of LM test solution and after 2 hours, 2 ml of urine were removed from collection bag; then collection was continued up to 5 hours. After 2 and 5 hours, total urine volume was measured and recorded. 1–2 drops of thimerosal (Sigma Aldrich) were added and samples were stored in a -80°C freezer until lactulose and mannitol concentration could be determined independently by HPAE employing pulsed amperometric detection (PAD) and LC-MSMS platform.

### HPAE-PAD

The carbohydrate analyzer HPAE-PAD system (Dionex Co., Sunnyvale, CA, USA) was composed of the following modules: ICS-5000 SP gradient pump, AS-DV automated Sampler for injections, and ED40 Electrochemical Detector (pulsed amperometric detector with gold on PTFE working electrode). A CarboPac MA-1 anion-exchange column (4x250 mm) with an associated CarboPac MA-1 guard column (4x50 mm), also from Dionex, was used as well Elution of the monosaccharide and disaccharide was achieved with an isocratic eluent of 450 mM NaOH at a flow-rate of 0.4 ml/min using ICS-5000 SP and MA-1 CarboPac Column. Column temperature was ambient. A 25-μl volume of each sample was injected automatically using the AS-DV Sampler. 5 levels of calibration standards were run with each batch to generate a standard curve from where results were automatically calculated using Chromeleon software.

#### Sample preparation

Frozen urine samples were thawed, vortexed for 10 seconds, and centrifuged for 10 minutes at 4500 rcf. After the vortex mixed the sample, 100 μl of supernatant urine and 50 μl of a solution containing melibiose (3.0 mM) were diluted in 2.85 ml of distilled de-ionized water. After vortex, 0.5 ml of the diluted sample was transferred to a sample vial with a filter cap and placed in the auto-sampler, from where samples were automatically injected into the HPAE system.

### LC-MSMS

The HPLC-MSMS system was composed of a LC- Shimadzu prominence LC20AD (Shimadzu, Maryland), with an ultramino 3 um 150 x 2.1 mm 100A aminopropylsilaneRestek column (Restek, Bellafonte, PA) with a flow rate of 0.30 ml/min with degasser and auto-sampler SIL-20AC HT (Shimadzu, Maryland), and column oven CTO-20A (Shimadzu, MD) run at 40°C. Mobile phase A was HPLC grade water with 0.1% formic acid and phase B was acetonitrile with 0.1% formic acid. Elution was programmed to start at 70% phase B for 0.5 minutes, then fall to 5% B at 2 minutes, return to 70%B at 2.5 minutes and equilibrate for two minutes prior to the next sample injection. MS/MS-API 5500 (AB Sciex, US) with turbo-ion probe (ESI) operated at 600C. The transitions monitored described before are listed with the retention times in Table 1 [17].

**Table 1:** Retention time in minutes (RT), dispersion potential (DP), entrance potential (EP), collision energy (CE), collision cell exit potential (CXP) Q1, Q3 in Thompson(Th) for ABSciex 5500 Qtrap MS/MS.

Analyte	Q1 (Th)	Q2 (Th)	RT (min)	DP	EP	CE	CXP
Mannitol 1	180.9	88.8	2.8	-150	-10	-20	-10
Mannitol 2	180.9	100.8	2.8	-150	-10	-14	-10
Mannitol 15C6	186.9	104.9	2.8	-150	-10	-20	-10
Lactulose 1	341.0	100.9	3.5	-120	-10	-20	-10
Lactulose 2	341.0	160.7	3.5	-120	-10	-14	-10
Lactulose 13C12	353.0	104.8	3.5	-120	-10	-20	-10

#### Reagents and calibrators

Calibrator solutions were prepared via serial dilutions of 10 mg/ml mannitol and lactulose (Sigma, St Louis MO), diluted in HPLC grade water. Internal standards contained 0.01 mg/ml of mannitol ^13^C6 and lactulose ^13^C12 (Sigma-Aldrich, St. Louis, MI). Pooled blank urine (urine collected prior to the administration of lactulose and mannitol) was spiked with independently prepared solutions containing serial dilutions of 10 mg/ml for assay validation and for quality control, on all runs. Linear responses in the calibration equation were observed with an r>0.999 in the range of 10–4000 μg/ml for both lactulose and mannitol with 95% confidence intervals of 2%. A second transition was monitored for each analyte to confirm identity and required to be within 20% of the relative peak area of the first transition used for quantification.

#### Sample preparation

Each urine sample was thawed and vortexed for 10 seconds. A volume of 20μl of sample was diluted in a mix of 70% acetonitrile containing internal standard 0.1% sodium acetate and diluted 1:4 in MS grade water; then transferred to the auto-sampler vial and vortexed for 10 seconds.

### Data analysis and calculation

Data was entered and analyzed using SPSS for Windows (version 10.5; SPSS Inc., Chicago, IL, USA). The WHO growth standard (2006) was used to calculate weight-for-age z-score (WAZ). Because the lactulose: mannitol results differ significantly from normal distribution, to compare the results between 2 and 5 hour urine collection, HPAE-PAD and LC-MSMS non-parametric t-tests were used.

Calculation of percent recovery of each sugar in the urine and their ratio:

PercentLactulose/mannitolrecovery=[{totalurinevolume(dL)overfirst2or5hoursXlactulose/mannitolconcentration(mg%)inurine}/Amount(mg)oflactulose/mannitoltaken]X100

Lactulosetomannitolratio=Percentlactuloserecovery/percentmannitolrecovery.

## Results

104 pairs (2 hours and corresponding 5 hours) of child-urine specimens from 104 children (52 male and 52 female) were studied. The median age of children at specimen collection time was 15.0 months with a range from 6.9 to 25.8 months. Their mean WAZ score was -0.92. Table 2 **s**hows the mass of lactulose and mannitol measured in 2 and 5 hours urine collection. Though 5 times as much lactulose as mannitol were administered, there were higher amount of mannitol than lactulose recovered with both 2 hours and 5 hours urine collection.

**Table 2:** Summary of lactulose and mannitol excretion in 2 hours and 5 hours urine collection.

	2 hours			5 Hours		
	HPAE-PAD	LM-MSMS	P	HPAE-PAD	LM-MSMS	p
Lactulose recovery (mg%)	18.2569 (8.5191, 39.1398)	20.6350 (11.5050, 43.6275)	<0.001	21.4112 (15.1179, 39.2776)	26.5900 (16.1625, 41.1050)	<0.001
Lactulose recovery rate	0.0011 (0.0005, 0.0019)	0.0013 (0.0006, 0.0022)	<0.001	0.0033 (0.0023, 0.0056)	0.0043 (0.0028, 0.0062)	<0.001
Percent lactulose recovery	0.1098 (0.0505, 0.1943)	0.1283 (0.0601, 0.2228)	<0.001	0.3279 (0.2284, 0.5597)	0.4253 (0.2770, 0.6229)	<0.001
Mannitol recovery (mg%)	34.0122 (10.8067, 84.8796)	37.2250 (10.7975, 85.9950)	0.151	46.0277 (23.8577, 74.0090)	49.8000 (25.8175, 78.3700)	<0.001
Mannitol recovery rate	0.0084 (0.0022, 0.0242)	0.0101 (0.0024, 0.0245)	0.127	0.0343 (0.0205, 0.0550)	0.0368 (0.0214, 0.0575)	<0.001
Percent mannitol recovery	0.8411 (0.2165, 2.4176)	1.0072 (0.2450, 2.4532)	0.127	3.4347 (2.0514, 5.5015)	3.6757 (2.1383, 5.7474)	<0.001
Lactulose to mannitol ratio	0.1167 (0.0742, 0.2548)	0.1498 (0.0723, 0.2915)	0.105	0.1104 (0.0697, 0.1648)	0.1172 (0.0721, 0.1845)	0.032

Data are expressed as median (inter quartile)

### Comparison between 2 and 5 hours urinary lactulose: Mannitol when measured by HPAE-PAD

**Fig 1:**
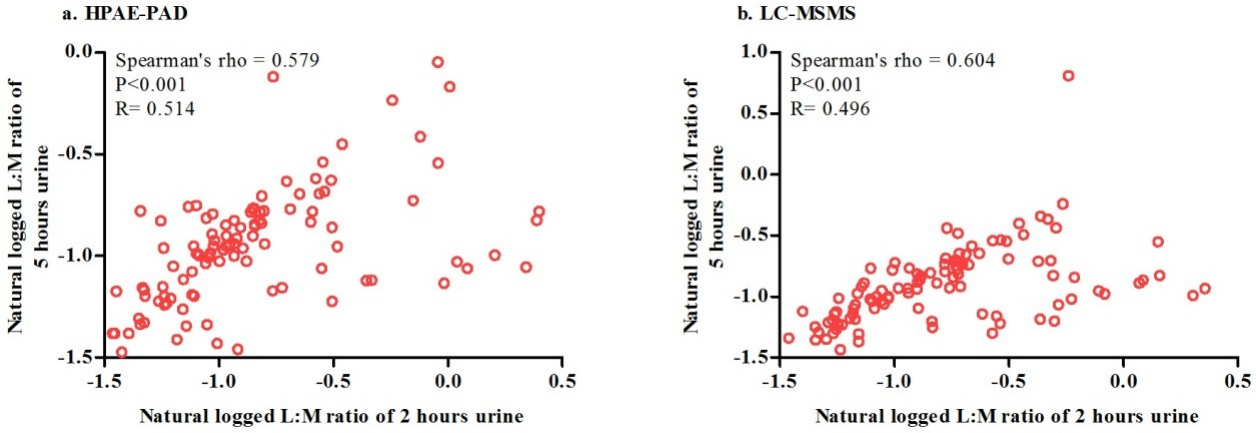
Correlations between standard 5 hours L:M ratio and shortened 2 hours L:M ratio. (a) HPAE-PAD and (b) LC-MSMS. (a) HPAE-PAD (n = 104); X axis indicates natural logged L:M ratio of 2 hours urine; Y axis stands for natural loged L:M ratio of 5 hours urine. (b) LC-MSMS (n = 104); X axis indicates natural logged L:M ratio of 2 hours urine; Y axis denotes natural loged L:M ratio of 5 hours urine. R stands for linear reression value; Spearman’s rho stands for Spearman’s nonparametric correlations.

Due to longer collection time, significantly higher percentage of lactulose (P<0.001) and mannitol (P<0.001) recovery (data are shown in Table 2) were found in 5 hours urine collection than in the corresponding 2 hours, but there was significant correlation between 2 hours lactulose recovery and 5 hours lactulose recovery (Spearman’s rho 0.548; P<0.001) and also significant correlation between 2 hours mannitol recovery and 5 hours mannitol recovery (Spearman’s rho 0.537; P<0.001). When these recovery were expressed as lactulose to mannitol ratio there was no significant difference between 2 and 5 hours urine collection (P = 0.138). LMR in 2 hours urine collection correlated well (Spearman’s rho 0.579; P<0.001) with LMR in traditional 5 hours urine collection. Fig 1 shows the relationship between LMR from 5 hours urine collection and shortened 2 hours urine collection.

### Comparison between 2 and 5 hours urinary lactulose: Mannitol when measured by LC-MSMS

Higher percentage of lactulose (P<0.001) and mannitol (P<0.001) recovery (data are shown in Table 2) were found in 5 hours urine collection than in the corresponding 2 hours, but there was significant correlation between 2 hours lactulose recovery and 5 hours lactulose recovery (Spearman’s rho 0.595; P<0.001) and also 2 hours mannitol recovery and 5 hours mannitol recovery (Spearman’s rho 0.500; P<0.001). When these recovery were expressed as lactulose to mannitol ratio there was no significant difference between 2 and 5 hours urine collection (P = 0.099). LMR in 2 hours urine collection correlated well (Spearman’s rho 0.604; P<0.001) with LMR in traditional 5 hours urine collection. Fig 1 shows the relationship between LMR from 5 hours urine collection and shortened 2 hours urine collection.

### Comparison between HPAE-PAD and LC-MSMS (Table 2)

LC-MSMS measured significantly higher concentration of lactulose when compared to HPAE-PAD (P<0.001 in both 2 hours and 5 hours urine), but there were significant correlations between lactulose concentrations measured by these methods in 2 hours (Spearman’s rho 0.870; P<0.001; R^2^ = 0.927) and 5 hours (Spearman’s rho 0.867; P<0.001; R2 = 0.904) urine.

LC-MSMS also measured higher concentration of mannitol than HPAE-PAD in 2 hours (P = 0.151) and 5 hours (P<0.001) urine, but mannitol concentrations measured by these methods had excellent correlation in 2 hours (Spearman’s rho 0.991; P<0.001; R^2^ = 0.980) and 5 hours (Spearman’s rho 0.981; P<0.001; R^2^ = 0.978) urine.

**Fig 2:**
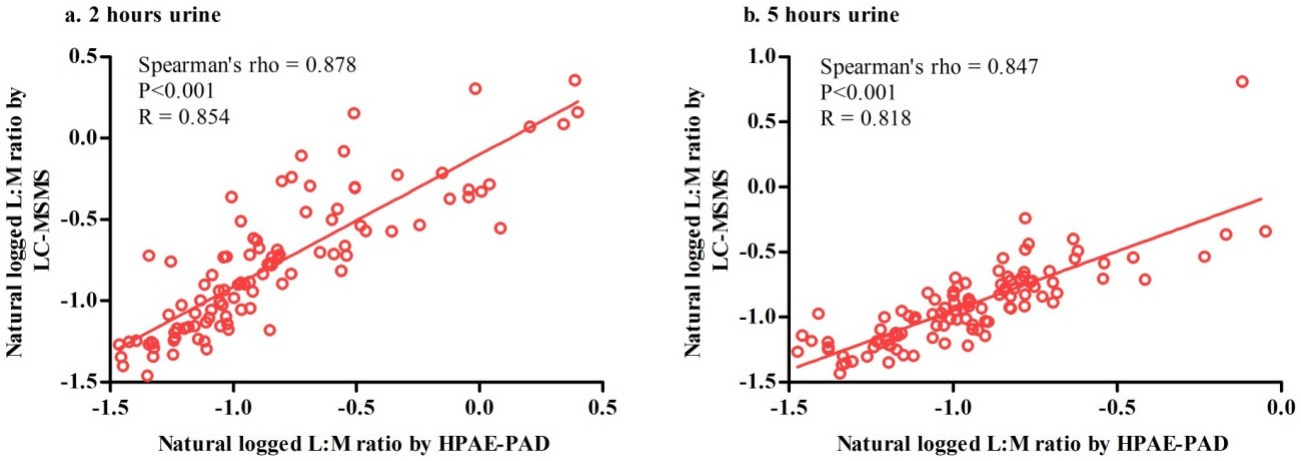
Correlation between 2 methods: HPAE-PAD and LC-MSMS. (a) 2 hours urine collection and (b) 5 hours urine collection. (a) 2 hours urine collection (n = 104); X axis indicates natural logged L:M ratio measured by HPAE-PAD; Y axis stands for natural loged L:M ratio measured by LC-MSMS (b) 5 hours urine collection (n = 104); X axis indicates natural logged L:M ratio measured by HPAE-PAD; Y axis denotes natural loged L:M ratio measured by LC-MSMS. R stands for linear reression value; Spearman’s rho stands for Spearman’s nonparametric correlations.

When the results were expressed as lactulose to mannitol ratio, LC-MSMS gave higher LMR; in 5 hours urine the difference was significant (P = 0.032) but in 2 hours urine the difference was not significant (P = 0.105). There were significant correlations between LM ratios from HPAE-PAD and LC-MSMS method for both 2 hours (Spearman’s rho 0.878; P<0.001; R2 = 0.623) and 5 hours (Spearman’s rho 0.847; P<0.001; R2 = 0.588) urine collection. Fig 2 shows the relationship between LMR by these two methods.

### Sensitivity and specificity of 2 hours urinary LMR, assuming 5 hours urinary LMR as standard

Most commonly, LM test results are used to categorize study population into normal and abnormal intestinal permeability depending on a cutoff value. In our study, we used LMR 0.09 [3] as the cutoff.

#### HPAE-PAD

In 5 hours urine collection, out of 104 children, 67 were categorized as abnormal and 37 as normal intestinal permeability. In case of 2 hours urine collection, among these 67 abnormal children, 56 children were identified as abnormal and among 37 normal children 26 children were identified as normal. Considering LMR from 5 hours urine collection as standard, LMR from 2 hours urine collection have sensitivity and specificity 83.58% and 70.27% respectively.

#### LC-MSMS

In 5 hours urine collection, out of 104 children, 69 were categorized as abnormal and 35 as normal. In case of 2 hours urine collection, among these 69 abnormal children, 59 children were identified as abnormal and among 35 normal children 24 children were identified as normal. Considering LMR from 5 hours urine collection as standard, LMR from 2 hours urine collection have sensitivity and specificity 85.51% and 68.57% respectively.

### Sensitivity and specificity of 5 hours urine, assuming 2 hours urine as standard

#### HPAE-PAD

In 2 hours urine collection, out of 104 children, 67 were categorized as abnormal and 37 as normal. In case of 5 hours urine collection, among these 67 abnormal children, 56 children were identified as abnormal and among 37 normal children 26 children were identified as normal. Considering L:M ratio from 2 hours urine collection as standard, L:M ratio from 5 hours urine collection have sensitivity and specificity 83.58% and 70.27% respectively.

#### LC-MSMS

In 2 hours urine collection, out of 104 children, 70 were categorized as abnormal and 34 as normal. In case of 5 hours urine collection, among these 70 abnormal children, 59 children were categorized as abnormal and among 34 normal children 24 children were identified as normal. Considering LMR from 2 hours urine collection as standard, LMR from 5 hours urine collection have sensitivity and specificity 84.29% and 70.59% respectively.

### Sensitivity and specificity of 2 hours urinary LMR with simulated cutoff, considering 5 hours urinary LMR as standard

LMR 0.09 is the most commonly used cutoff value to categorize study population into normal and abnormal intestinal permeability. This cutoff was derived from 5 hours urine collection, and may not be appropriate for 2 hours urine collection. Using linear regression model and LMR 0.09 as reference cutoff for 5 hours urine collection, we calculated the corresponding cutoff value for 2 hours urine collection. Our calculated cutoff values for 2 hours urine collection are 0.075 and 0.070 for HPAE-PAD and LC-MSMS respectively.

#### HPAE-PAD

In 5 hours urine collection considering 0.09 as cutoff, 67 children were categorized as abnormal and 37 children as normal intestinal permeability. In case of 2 hours urine collection applying 0.075 as cutoff, among these 67 abnormal intestinal permeability children, 64 children were identified as abnormal and among 37 normal children 22 children were identified as normal intestinal permeability. Considering LMR from 5 hours urine collection as standard, LMR from 2 hours urine collection have sensitivity and specificity 95.52% and 59.46% respectively.

#### LC-MSMS

In 5 hours urine collection, 69 children were categorized as abnormal and 35 children as normal intestinal permeability. In case of 2 hours urine collection applying 0.070 as cutoff, among these 69 abnormal intestinal permeability children, 67 children were identified as abnormal and among 35 normal children 23 children were identified as normal intestinal permeability. Considering LMR from 5 hours urine collection as standard, LMR from 2 hours urine collection have sensitivity and specificity 97.10% and 65.71% respectively.

### Comparison between male and female children

When the results were compared between male and female children, female children had significantly higher lactulose to mannitol ratio for both 2 and 5 hours urine collection and both assay methods (data shown in Table 3). There were no significant difference in percent lactulose recovery between male and female children for both 2 and 5 hours urine collection. In the case of percent mannitol recovery, male children had higher percent recovery, but the differences were only significant for 5 hours urine collection.

**Table 3:** Lactulose mannitol test results of male and female children.

	2 hours urine collection						5 hours urine collection					
	HPAE-PAD			LC-MSMS			HPAE-PAD			LC-MSMS		
	% lactulose recovery	% mannitol recovery	L:M ratio	% lactulose recovery	% mannitol recovery	L:M ratio	% lactulose recovery	% mannitol recovery	L:M ratio	% lactulose recovery	% mannitol recovery	L:M ratio
Male N = 52	0.1157	1.5007	0.0946	0.1233	1.5045	0.1091	0.3556	4.1304	0.0982	0.4253	4.4813	0.0962
Female N = 52	0.1001	0.7275	0.1412	0.1369	0.7739	0.1861	0.3147	2.7392	0.1316	0.4088	3.0678	0.1452
P-Value	0.897	0.125	0.021	0.581	0.156	0.015	0.640	0.005	0.009	0.933	0.004	0.003

Data are median where applicable

## Discussion

As far as we know, this is the first study where a large number of healthy children were included to depict intestinal permeability in terms of 2 and 5 hours urinary lactulose and mannitol recovery. In our study we were able to show that 5 hours urine collection can be replaced by 2 hours urine collection, which is also supported by a previous study [18] conducted on adult patients. The test result was expressed as the rate recovery of ingested lactulose divided by the rate recovery of ingested mannitol. Shortened collection time gave decreased recovery rate of these sugars but the ratio remained unchanged. The LMR can only be affected by the difference in timing of the excretion of lactulose and mannitol. Peak urinary recovery of both lactulose and mannitol occur after 7 to 9 hours of LM solution administration [19]. If we see, how many numbers of children are detected as normal and abnormal intestinal permeability for 2 and 5 hours urine collection? Considering more commonly used 5 hour urine collection as standard 2 hours urine collection gives better sensitivity (83–85%) and specificity (68–70%) by both HPAE-PAD and LC-MSMS method. Similar sensitivity and specificity also found for 5 hours, considering 2 hours urine collection as standard. In this study, we derived the cutoff value for 2 hours urine collection from the cutoff value of 5 hours urine collection using mathematical simulation. So far, there is no defined cutoff value for 2 hours urine collection. In future studies these cutoff values can be used as reference for 2 hours urine collection. We found applying these cutoffs increased the sensitivity (95–97%) and decreased specificity (59–66%) of 2 hours urine collection. A cutoff value yielding better sensitivity can be a better choice for surveillance study targeting intestinal permeability.

About 49.6% sugar molecules reach the colon within 2 hours of LM solution administration [20],indicating that in 2 hours LMR there is less contribution from colonic absorption compared to small intestinal absorption where these sugar molecules are predominately absorbed. With an increase in urine collection time, interference by colonic absorption of sugar molecules also increases. We consider assessment of small intestinal permeability with reduced colonic absorption better because colonic bacteria metabolize lactulose and mannitol molecules. Thus colonic absorption is not only dependent on colonic permeability, but also colonic environment. On the other hand, a shorter collection time than 2 hours gives very small percentage of sugar molecular recovery and may not be informative [19]. We do not know the exact reason for increased lactulose to mannitol ratio in female children. There’s no significant difference between male and female children in terms of factors affecting L:M ratio: age or weight-for-age z-score. This may be due to hormonal influence. It is known that thyroid hormone [21], progesterone [22], testosterone [23] imbalance can promote leaky gut. However; further studies are required to resolve this question.

Two methods (HPAE-PAD and LC-MSMS) are compatible as both measure recovery rate of lactulose and mannitol very efficiently at 2 hours and 5 hours with r value 0.85 to 0.99 for lactulose, mannitol and L:M ratio. Therefore, any of these two can be used in practice. However, we should recommend the cost-effective and user-friendly one. We assessed intestinal permeability after the ingestion of lactulose and mannitol sugar solutions in children aged 6.9 to 25.8 months. In our study, the urine samples were collected at 2 hours and 5 hours after the ingestion of these sugar solutions and found similar results following a 2 hour and 5 hour urine collection (the L:M ratio remained unchanged), however a 5-hour urine collection at present considered as a standard practice for L:M ratio measurement but we considered the 2 hour collection for avoiding possible inconveniences of the enrolled child participants. The notable inconvenience includes the need of the family members staying long period of time in the clinics/diagnostic places with their children. The shortened collection time of 2 hours would be convenient for them because the time and consequently, the stay of partipants family members at the collection sites are reduced more than half of it’s conventional time (5 hours). As L:M ratio is almost same in both conventional 5 hours and shortened 2 hours urine sample collections, we believe that a shorter time (i.e 2 hours) collection would be suitable for the children and family members for the assessment of intestinal permeability using these sugar solutions. This may be helpful to introduce this assay in clinical practice by the paedtricians.

## Supporting information

S1 Data (XLSX)
